# Supplementary material for: Selection of Stable Reference Genes for Gene Expression Studies in Activated and Non-Activated PBMCs Under Normoxic and Hypoxic Conditions
Source: Int J Mol Sci. 2025 Jul 15;26(14):6790. doi: 10.3390/ijms26146790 (PMC12294867; doi:10.3390/ijms26146790)
Supplement: Supplementary file 1 [file ijms-26-06790-s001.zip › ijms-3688217-supplementary.pdf]

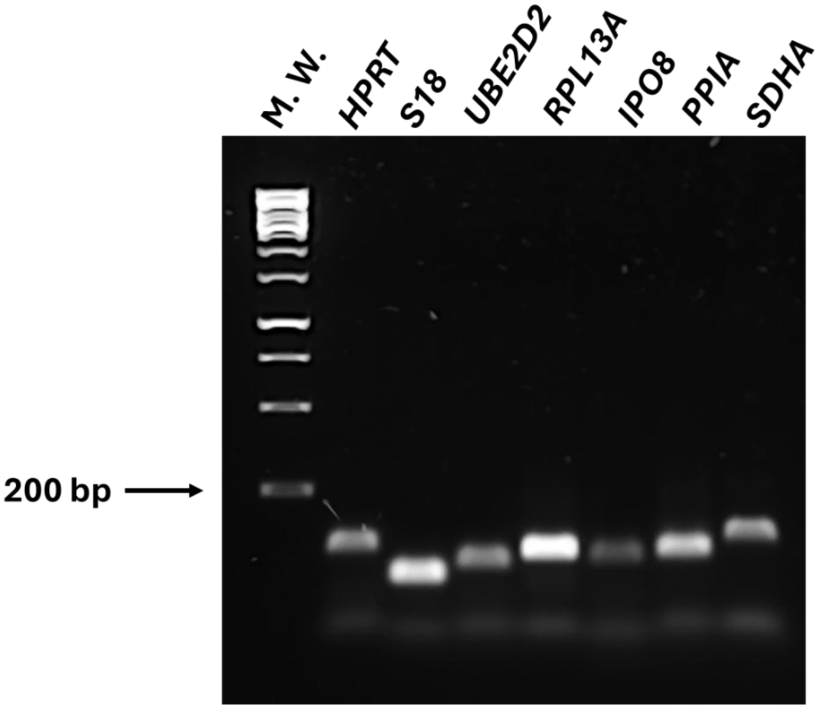

Figure S1. Nucleic acid gel electrophoresis and visualization. A representative image showing the distribution of amplification products for each reference gene. The molecular weight (M. W.) marker was also loaded into the gel to check the size in base pairs (bp) of DNA fragment signals.

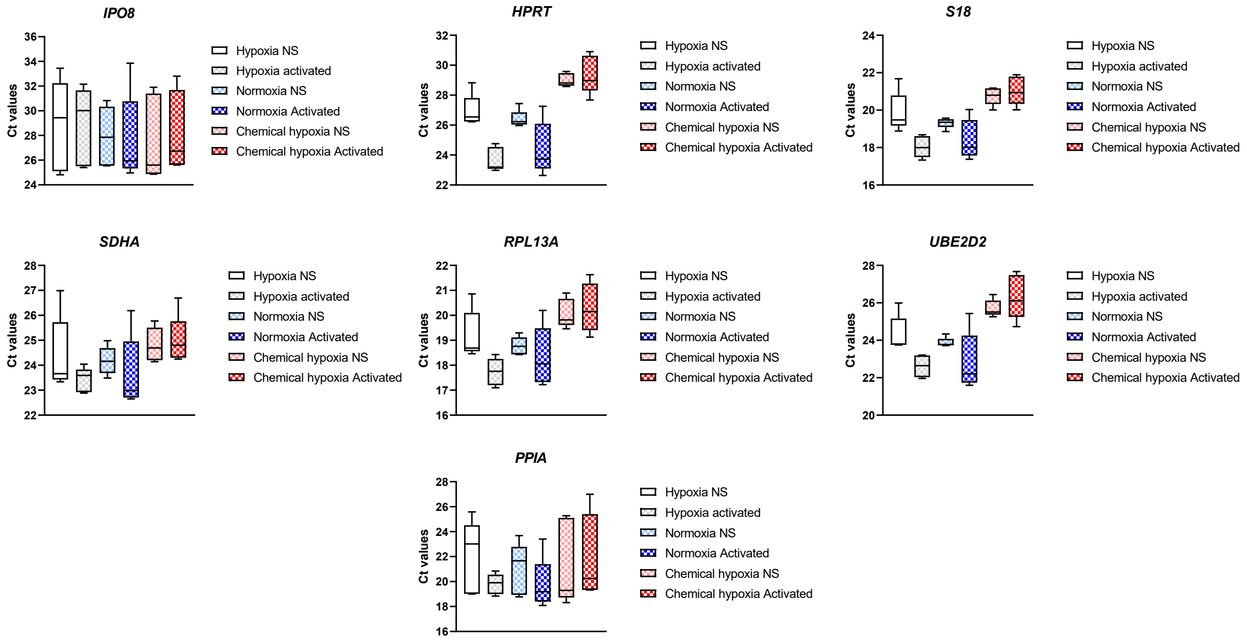

Figure S2. Ct values for seven candidate reference genes stratified by experimental groups. The boxes represent the upper and the lower quartiles of cycle thresholds range with medians. Whiskers extend to the smallest and largest values. NS denotes non-stimulated cells.

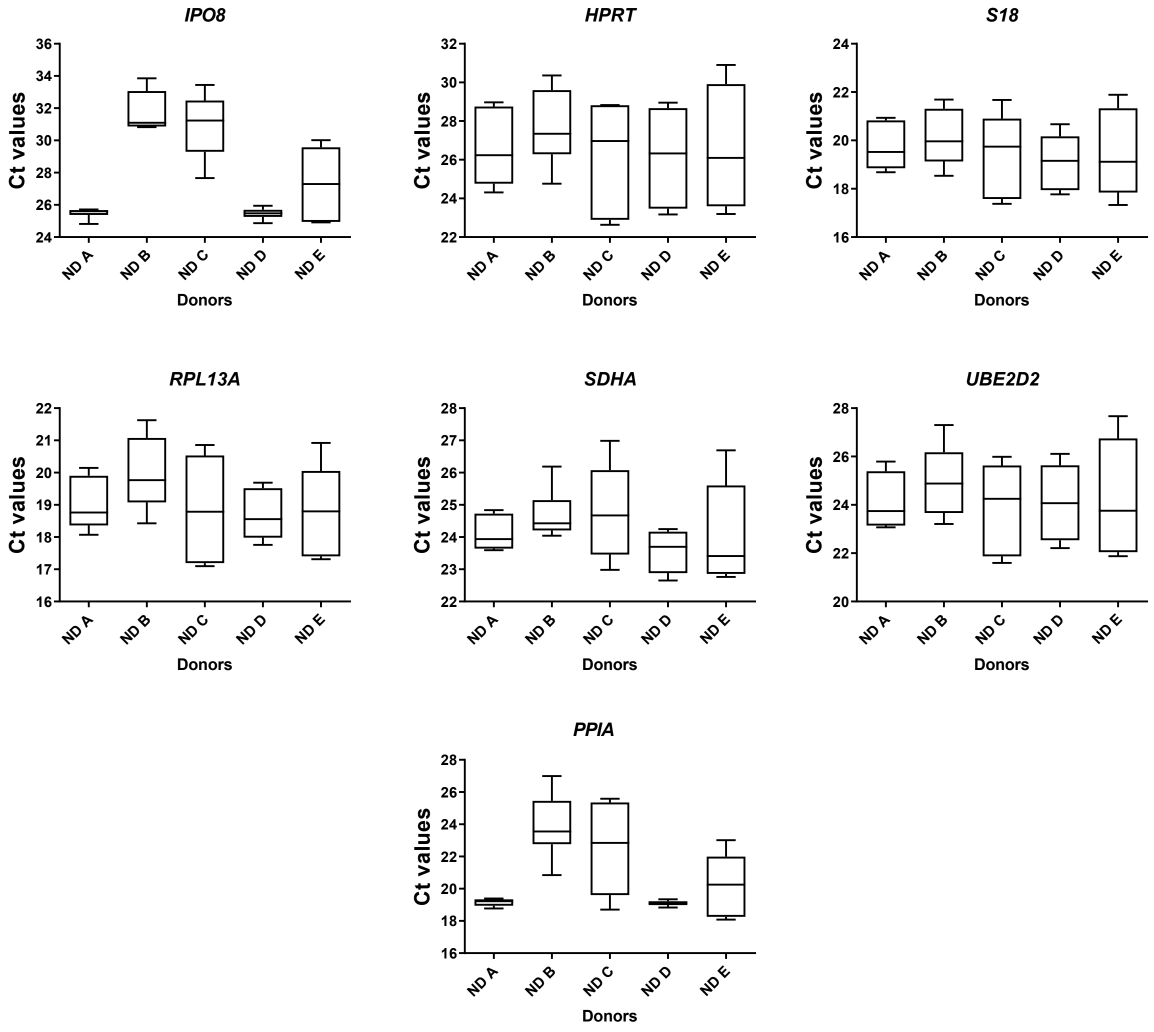

Figure S3. Ct values for seven candidate reference genes presented separately for each individual donor for all groups. The boxes represent the upper and the lower quartiles of cycle thresholds range with medians. Whiskers extend to the smallest and largest values.

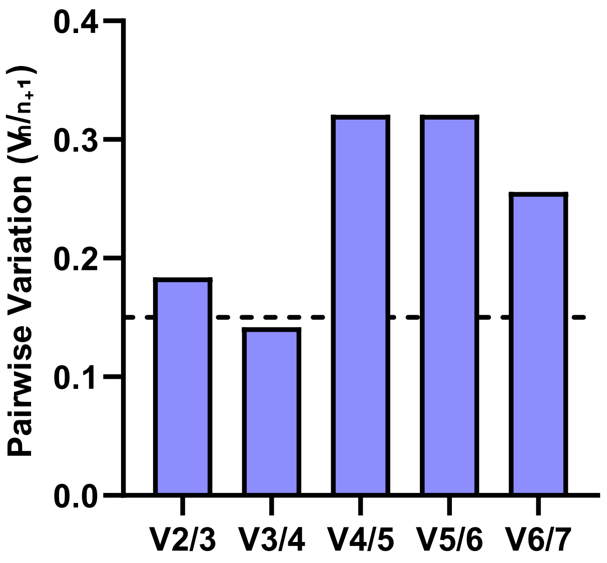

Figure S4. Determination of the optimal number of reference genes for qPCR normalization based on pairwise variation (V) values calculated using the geNorm algorithm. The pairwise variation $V_{3/4}$ dropped below the commonly accepted threshold of 0.15, indicating that the inclusion of a fourth gene does not significantly improve normalization.

Table S1. Expression stability of candidate reference genes across all samples evaluated using Coefficient of Variation (CV).

Gene	CV [%]	Ranking
SDHA	4.56	1
RPL13A	6.26	2
UBE2D2	6.73	3
S18	6.77	4
HPRT	8.90	5
IPO8	10.78	6
PPIA	12.15	7

Table S2. Comparison of candidate reference genes based on ∆Ct method. Mean ∆Ct values represent the average differences in expression between gene pairs. Standard deviation (SD) reflects the variability in delta Ct values across samples, based on which the variation in gene expression differences can be assessed.

**Genes**	Mean Ct	SD	Mean SD
IPO8* vs *HPRT	-1.56	3.59	2.86
IPO8* vs *S18	-8.51	3.31	
IPO8* vs *SDHA	-3.95	2.76	
IPO8* vs *RPL13A	-9.12	2.89	
IPO8* vs *UBE2D2	-3.91	3.22	
IPO8* vs *PPIA	-7.21	1.59	
HPRT* vs *IPO8	1.56	3.59	1.86
HPRT* vs *S18	-7.06	1.13	
HPRT* vs *SDHA	-2.39	1.68	
HPRT* vs *RPL13A	-7.57	1.32	
HPRT* vs *UBE2D2	-2.36	0.87	
HPRT* vs *PPIA	-5.65	2.58	
S18* vs *IPO8	8.62	3.11	1.36
S18* vs *HPRT	7.06	1.13	
S18* vs *SDHA	4.67	0.77	
S18* vs *RPL13A	-0.50	0.41	
S18* vs *UBE2D2	4.71	0.50	
S18* vs *PPIA	1.41	2.26	
SDHA* vs *IPO8	3.95	2.76	1.53
SDHA* vs *HPRT	2.39	1.68	
SDHA* vs *S18	-4.67	0.77	
SDHA* vs *RPL13A	-5.17	0.74	
SDHA* vs *UBE2D2	0.04	1.01	
SDHA* vs *PPIA	-3.26	2.21	
RPL13A* vs *IPO8	9.12	2.89	1.34
RPL13A* vs *HPRT	7.57	1.32	
RPL13A* vs *S18	0.50	0.41	
RPL13A* vs *SDHA	5.17	0.74	
RPL13A* vs *UBE2D2	5.21	0.66	
RPL13A* vs *PPIA	1.92	2.04	
UBE2D2* vs *IPO8	3.91	3.22	1.44
UBE2D2* vs *HPRT	2.36	0.87	
UBE2D2* vs *S18	-4.71	0.50	
UBE2D2* vs *SDHA	-0.04	1.01	

UBE2D2 vs *RPL13A*	-5.21	0.66	
UBE2D2 vs *PPIA*	-3.29	2.36	
PPIA vs *IPO8*	7.21	1.59	2.17
PPIA vs *HPRT*	5.65	2.58	
PPIA vs *S18*	-1.41	2.26	
PPIA vs *SDHA*	3.26	2.22	
PPIA vs *RPL13A*	-1.92	2.04	
PPIA vs *UBE2D2*	3.29	2.36	

Table S3. Pearson correlation matrix showing pairwise relationships among candidate reference genes based on raw Ct values. The correlation coefficients (r) and corresponding p-values are presented for each gene pair. Strong and statistically significant correlations ($p < 0.05$) suggest co-expression stability. The lower panel summarize the correlation of each gene with the BestKeeper index, indicating the consistency of individual genes with the overall stability profile.

Pearson correlation coefficient (r)							
**vs.**	**HKG 1 (*IPO8*)**	**HKG 2 (*HPRT*)**	**HKG 3 (*S18*)**	**HKG 4 (*SDHA*)**	**HKG 5 (*RPL13A*)**	**HKG 6 (*UBE2D2*)**	**HKG 7 (*PPIA*)**
**HKG 2**	0,135	-	-	-	-	-	-
**p-value**	0,477	-	-	-	-	-	-
**HKG 3**	0,163	0,972	-	-	-	-	-
**p-value**	0,392	0,001	-	-	-	-	-
**HKG 4**	0,425	0,763	0,815	-	-	-	-
**p-value**	0,019	0,001	0,001	-	-	-	-
**HKG 5**	0,315	0,936	0,952	0,793	-	-	-
**p-value**	0,089	0	0,001	0,001	-	-	-
**HKG 6**	0,153	0,972	0,964	0,796	0,939	-	-
**p-value**	0,419	0,001	0,001	0,001	0,001	-	-
**HKG 7**	0,853	0,452	0,468	0,499	0,618	0,434	-
**p-value**	0,001	0,012	0,009	0,005	0,001	0,017	-
**BestKeeper vs. coeff. of corr. [r]**	HKG 1	HKG 2	HKG 3	HKG 4	HKG 5	HKG 6	HKG 7
	0,627	0,841	0,857	0,831	0,922	0,841	0,833
**p-value**	0,001	0,001	0,001	0,001	0,001	0,001	0,001

Table S4. Linear regression analysis of candidate reference genes relative to the BestKeeper index. The table presents the Pearson correlation coefficient (r), coefficient of determination (r^2), intercept and slope of the regression line, standard error (SE) of the slope, p-values, and relative expression power (x-fold). All genes showed statistically significant correlations with the BestKeeper index (p=0.001), with *RPL13A* demonstrating the highest correlation. Expression power reflects the dynamic range of gene expression.

Regression Analysis: HKG vs. BestKeeper							
	IPO8	*HPRT*	*S18*	*SDHA*	*RPL13A*	*UBE2D2*	*PPIA*
**coeff. of corr. [r]**	0,63	0,84	0,86	0,83	0,92	0,84	0,83
**coeff. of det. [r^2]**	0,39	0,71	0,73	0,69	0,85	0,71	0,69
**intercept [CP]**	-1,31	-4,21	2	10,01	2,02	3,03	-11,9
**slope [CP]**	1,28	1,34	0,76	0,62	0,74	0,92	1,43
**SE [CP]**	±2,41	±1,305	±0,695	±0,626	±0,471	±0,9	±1,436
**p-value**	0,001	0,001	0,001	0,001	0,001	0,001	0,001
**power of HKG [x-fold]**	**2,15**	**3**	**1,75**	**1,53**	**1,65**	**1,76**	**3,23**

Table S5. Classification of reference gene candidates based on stability values performed using the RefFinder algorithm.

Ranking Order (Better-Good-Average)							
**Method**	1	2	3	4	5	6	7
**ΔCt**	*RPL13A*	*S18*	*UBE2D2*	*SDHA*	*HPRT*	*PPIA*	*IPO8*
**BestKeeper**	*SDHA*	*RPL13A*	*S18*	*UBE2D2*	*HPRT*	*PPIA*	*IPO8*
**NormFinder**	*RPL13A*	*S18*	*SDHA*	*UBE2D2*	*HPRT*	*PPIA*	*IPO8*
**GeNorm**	*S18/RPL13A*	-	*UBE2D2*	*SDHA*	*HPRT*	*PPIA*	*IPO8*
**Recommended comprehensive ranking**	***RPL13A***	***S18***	***SDHA***	***UBE2D2***	***HPRT***	***PPIA***	***IPO8***

Table S6. Ranking of candidate reference genes under hypoxic conditions based on stability values calculated using the RefFinder algorithm.

Ranking Order (Better-Good-Average)							
**Method**	1	2	3	4	5	6	7
	SDHA	*RPL13A*	*UBE2D2*	*S18*	*PPIA*	*HPRT*	*IPO8*
**Recommended comprehensive ranking**	***RPL13A***	***UBE2D2***	***SDHA***	***S18***	***HPRT***	***PPIA***	***IPO8***

Table S7. Ranking of candidate reference genes under normoxic conditions based on stability values calculated using the RefFinder algorithm.

Ranking Order (Better-Good-Average)							
**Method**	1	2	3	4	5	6	7
**ΔCt**	*UBE2D2*	*SDHA*	*RPL13A*	*S18*	*HPRT*	*PPIA*	*IPO8*
**BestKeeper**	*RPL13A*	*S18*	*SDHA*	*UBE2D2*	*HPRT*	*PPIA*	*IPO8*
**NormFinder**	*SDHA*	*UBE2D2*	*RPL13A*	*HPRT*	*S18*	*PPIA*	*IPO8*
**GeNorm**	*S18/RPL13A*	-	*UBED2D*	*SDHA*	*HPRT*	*PPIA*	*IPO8*
**Recommended comprehensive ranking**	***RPL13A***	***UBE2D2***	***SDHA***	***S18***	***HPRT***	***PPIA***	***IPO8***

Table S8. Ranking of candidate reference genes under chemically induced hypoxia, based on stability values calculated using the RefFinder algorithm.

	**Ranking Order (Better-Good-Average)**						
**Method**	1	2	3	4	5	6	7
**ΔCt**	*RPL13A*	*S18*	*HPRT*	*UBE2D2*	*SDHA*	*IPO8*	*PPIA*
**BestKeeper**	*S18*	*SDHA*	*RPL13A*	*HPRT*	*UBE2D2*	*IPO8*	*PPIA*
**NormFinder**	*RPL13A*	*S18*	*HPRT*	*UBE2D2*	*SDHA*	*IPO8*	*PPIA*
**GeNorm**	*HPRT/UBE2D2*	-	*S18*	*RPL13A*	*SDHA*	*IPO8*	*PPIA*
**Recommended comprehensive ranking**	***RPL13A***	***S18***	***HPRT***	***UBE2D2***	***SDHA***	***IPO8***	***PPIA***
